# The Effect of a Lecture‐Based Educational Intervention to Improve the Nutrition Knowledge and Behavior of Plant‐Based Seventh‐Day Adventists Living in the United Kingdom

**DOI:** 10.1002/hsr2.70440

**Published:** 2025-02-13

**Authors:** Robert K. Janko, Irmgard Haussmann, Ashok Patel

**Affiliations:** ^1^ Department of Health Sciences Birmingham City University Birmingham UK

**Keywords:** diet, nutrition, plant‐based diets, seventh‐day adventists, supplement

## Abstract

**Background and Aims:**

The Adventist lifestyle, which encourages the consumption of a vegetarian or vegan diet, has been associated with several health benefits; however, the nutrition knowledge of Adventists about essential nutrients in the context of plant‐based diets has not yet been assessed, therefore, this study evaluated the efficacy of an expert‐led lecture‐based educational intervention on the nutrition knowledge and habits of plant‐based Seventh‐day Adventists.

**Methods:**

The intervention, delivered in the form of an online lecture by a clinical nutrition expert, comprised a 30‐min lecture emphasizing the role of essential nutrients for vegans and vegetarians, the role of dietary supplements in COVID‐19 prevention. Nutritional knowledge was assessed by using a 25‐item questionnaire before and after the lecture, with a follow‐up survey administered 4 weeks after the lecture to examine changes in dietary behaviors and supplement use.

**Results:**

Thirty‐seven participants completed the study. The mean test scores significantly improved from 8.49 (SD 3.75) pre‐lecture to 20.03 (SD 2.99) post‐lecture (*p* < 0.001). Subsequent behavioral changes were reported, including increased supplement use and dietary modifications, underscoring the intervention's impact.

**Conclusion:**

This study highlights the effectiveness of a targeted educational interventions in improving nutritional knowledge among plant‐based Seventh‐day Adventists. Health promotion activities conducted by the church should aim to inform church members of the need for well‐planned plant‐based diets and of the importance of appropriate supplementation.

## 1 Introduction

The health benefits of the Adventist lifestyle include a lower risk of chronic diseases and mortality [1]. The Adventist church advocates for a healthy lifestyle which include abstinence from alcohol and smoking and the consumption of a healthy and mostly vegetarian diet [2].

In a survey including over 63,000 Adventists across the globe, 19% reported consuming a vegetarian or vegan diet [2].

Although Adventists place a large emphasis on diet, their use of dietary supplements may be less common than expected, especially among those following plant‐based diets. For instance, Thorpe et al. [3] showed that vegan women had a 55% greater risk of hip fracture among the cohort. In subgroup analysis, however, it was revealed that only those females who refrained from supplementing their dietary intake of vitamin D and calcium displayed an increased risk of bone fractures, highlighting the importance of using certain supplements on a vegan or vegetarian diet. Examining the nutrient intake of vegans in the Adventist Health Study 2 (AHS‐2) cohort, they were shown to have higher intakes of vitamin C, magnesium, fiber, folate, vitamin E, and β‐carotene from dietary sources, but the study showed that strict vegetarians were the least likely to use dietary supplements [4] which may be somewhat of a concern as nutrients such as vitamin B12 cannot be obtained from wholefood sources on a vegan diet [5] and should, therefore, be acquired through supplements or fortified foods.

Although the official position of the church is that vegan diets should be supplemented with vitamin B12, vitamin D, and potentially calcium [6], there could be a number of Adventists who may refuse to use supplements due to their belief concerning the nutritional adequacy of a wholefoods plant‐based diet and for fear of anything perceived as unnatural or synthetic.

Currently, there is not a uniform definition of a plant‐based diet in the scientific literature. According to a review, 50% of the studies that have used the term referred to a vegan diet, which excludes all animal products. On the other hand, the rest of the studies have used the term to define a diet without the regular use of meat products, which may include vegetarian diets, which allow for the consumption of dairy and eggs, pescatarian diets, that can include the consumption of fish, and semi‐vegetarian diets, which may allow the infrequent consumption of any kind of animal product [7]. For the purposes of this study, the term plant‐based was used to include vegan, vegetarian, or pescatarian diets.

Lecture‐based educational nutritional interventions have been shown to improve participants’ nutrition knowledge as confirmed by Cusack et al. [8]. Furthermore, a lecture‐based intervention delivered to pharmaceutical science students in Japan significantly improved the dietary supplement knowledge of participants [9]. Furthermore, a study involving American college students demonstrated that a lecture‐based intervention not only enhanced knowledge but also resulted in behavioral changes [10]. Another study involving Adventists showed that interventions that are aimed at changing participants’ attitudes towards diet and lifestyle successfully resulted in subsequent behavioral change [11]; however, the nutrition knowledge and belief of Adventists living in the United Kingdom have never been assessed, therefore, the objective of this study was to assess the impact of an expert‐led lecture‐based educational intervention, specifically tailored for vegan and vegetarian Seventh‐day Adventists on their nutritional knowledge and behavior.

## 2 Methods

### 2.1 Ethical Approval

This study received ethical approval from the Health, Education and Life Sciences Faculty Academic Ethics Committee of Birmingham City University.

### 2.2 Data Collection

Participants were recruited using convenience sampling and were eligible for inclusion if they were UK‐based and followed a vegan, vegetarian, or pescatarian diet. Anyone under 18 years of age was excluded from participation. In addition, Adventists living outside of the United Kingdom were excluded. The sample size for the study was calculated using the Statulator online statistical calculator for paired means [12]. The calculation aimed to detect an effect size of 0.5 with 80% power and a 5% significance level (two‐sided test). Based on this, a minimum of 34 paired observations was required to achieve the predetermined statistical power and significance level.

Social media platforms such as Adventist Facebook groups and Instagram were used for recruitment. Furthermore, vegan and vegetarian participants who had previously provided consent for a longitudinal study conducted by the authors were invited to participate in this study [13]. Anyone who indicated interest in participating was provided with the Participant Information Sheet and the consent form, which was to be returned to the lead author via email before the study launch.

### 2.3 Study Design

This study employed a quasi‐experimental design and collected cross‐sectional data before and after an educational intervention in the form of an online lecture. The primary outcome of this study was the change in test scores before and after the intervention. Before the lecture, participants were instructed to complete an online questionnaire via Microsoft Forms, aimed at evaluating their baseline knowledge and beliefs regarding diet and nutrition, both generally and in relation to the treatment and prevention of COVID‐19. Subsequently, participants were required to listen to the online lecture and were then asked to fill in the questionnaire again, to determine if the intervention had any impact on their nutritional knowledge. The presentation lasted approximately 30 min. Participants were provided with an email invite to a Zoom meeting, which they could access at the time of the event, during which they were not required to turn on their cameras.

The lecture incorporated information on the levels of evidence using the hierarchy pyramid [14], which provided a visual representation of the concept and covered various key areas including the advantages and any potential serious drawbacks of a vegan diet such as specific nutritional concerns like the risk of deficiencies in vitamins B12 and D, omega‐3 fatty acids and iron among vegans. Strategies for mitigating these risks through dietary planning and the use of supplementation were emphasized and it presented essential information about the role of specific supplements, such as zinc, vitamin C, curcumin, and probiotics, in the context of COVID‐19 disease prevention and treatment. The potential benefits of these supplements were highlighted based on the best available research evidence. Data from clinical trials and systematic reviews was utilized to highlight the dietary supplements that have been shown to reduce the risk of COVID‐19 infection or mitigate the severity of symptoms, including hospitalization, caused by the infection.

This educational intervention utilized the principles of the Health Belief Model as the theoretical framework. Accordingly, the intervention aimed to result in behavior change by influencing participants’ views and beliefs regarding susceptibility and potential barriers to appropriate nutrient intake [15]. Therefore, the lecture was designed to address and tackle common misconceptions and barriers held by some Adventists regarding the adequacy of plant‐based diets and the use of supplements, most importantly vitamin B12 and vitamin D, and highlighted the potential dangers of inappropriately planned plant‐based diets.

### 2.4 The Questionnaire

This study has been reported in line with the Checklist for Reporting Results of Internet E‐Surveys (CHERRIES) as published by [16].

The pre‐ and post‐intervention questionnaires developed for this study contained multiple‐choice and open‐ended questions. Twenty‐five test questions were used to evaluate nutritional knowledge. The follow‐up questionnaire was sent to participants 4 weeks after the lecture and gathered information about any potential changes in diet or supplement use as well as in beliefs and views about the topics discussed.

Validated through a small pilot involving 6 Adventists who were not sampled for the main study, the questionnaire demonstrated high internal consistency (Cronbach's alpha = 0.873) and test‐retest reliability (Pearson's *r* = 0.85, *p* = 0.03).

The highest score attainable in the nutrition knowledge test was 25. The questionnaire was composed of two main sections; one focused on general nutritional information with 11 questions with a total possible score of 11, and the second section of the questionnaire contained 14 questions that focused on the efficacy of nutritional supplements such as vitamin D, zinc, or curcumin in the treatment of COVID‐19 totaling a maximum score of 14.

### 2.5 Statistical Analysis

All statistical analysis was completed in SPSS version 29.0.1. 1. The paired samples *t*‐test was used to compare the pre‐ and post‐intervention scores. A *p*‐value of less than 0.05 was considered statistically significant and all tests were two‐sided. Cohen's d was calculated as an effect size was calculated by dividing the mean difference by the standard deviation of the mean difference. As the sample size was below 50, Hedges’ g was also calculated from Cohen's d by adjusting for small sample size bias using the below formula:

d × 1 − (3/4n − 9), where d denotes Cohen's d, and n denotes the sample size.

Effect sizes for both Cohen's d and Hedges’ g were considered as follows [17]:

0.2 = small effect,

0.5 = medium effect,

0.8 or above = large effect.

Corresponding 95% confidence intervals (CI) were calculated for the effect size and the differences in means and were considered statistically significant if the 95% CI did not cross 0.

Correlation coefficients (r) assessed the relationship between the pre‐ and post‐intervention scores; *r* ≥ 0.7 indicated a strong relationship, meaning consistent scoring by participants across both tests.

Repeated measures ANOVA was used to evaluate whether the change in scores was different between vegan, vegetarian, and pescatarian subjects with Games‐Howell for post hoc testing due to varying group sizes. With regards to the improvement in scores, potential covariates such as sex and education status were adjusted for using ANCOVA.

Changes in dichotomous variables were evaluated using the McNemar test. Potential associations between dichotomous variables were evaluated using the Chi‐square test of independence or Fisher's exact test for small, expected frequencies.

## 3 Results

In total, 37 participants were recruited in this study, 20 of whom identified as vegan, 13 as vegetarian, and 4 as pescatarian. Participants’ mean age in years was 45 (SD 12.9) and baseline assessment showed that four participants used vitamin B12 supplementation, 11 supplemented vitamin D, and 10 supplemented both nutrients. After the educational intervention, 18 participants confirmed having begun to use a new supplement at follow‐up. Additionally, 22 participants reported increasing their intake of fortified foods, while 2 individuals indicated consuming more fish or dairy. In the follow‐up questionnaire, only 3 individuals expressed skepticism about using dietary supplements, whereas 31 participants reported being more mindful of potential nutrient deficiencies in their diet and supplementing as needed.

**Figure 1:**
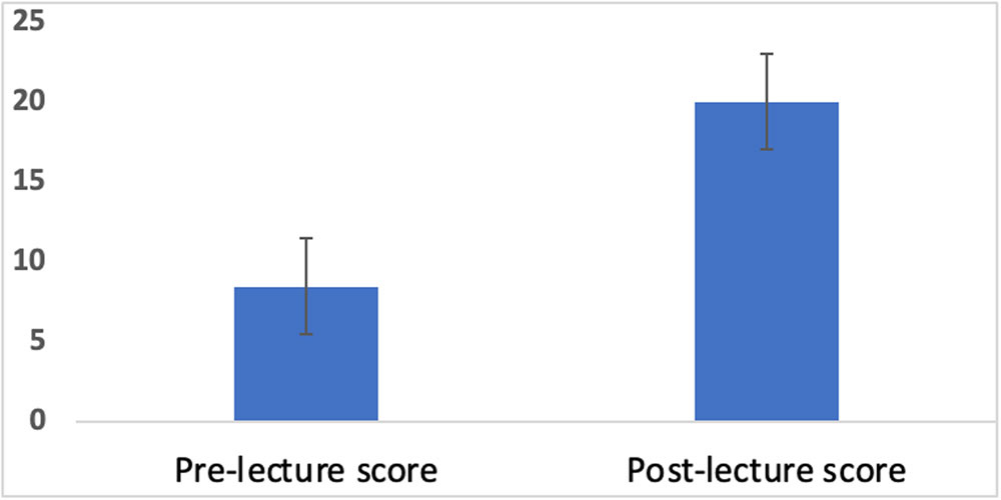
Pre‐ and post‐lecture test scores (mean, SD).

The mean test scores of participants before and after the lecture are presented in Figure 1. Post‐lecture mean test scores significantly improved from 8.49 (SD 3.75) to 20.03 (SD 2.99) (*p* < 0.001) with a large effect size (Cohen's *d* = −2.739, Hedges’ *g* = −2.681). Repeated measures ANOVA further confirmed the significant change in scores for all participants (Wilk's Lambda for test score, *p* < 0.001); however, it showed no differences in the test scores between the three diet groups (Wilk's lambda test score*diet followed, *p* = 0.090).

The correlation between the scores before and after the lecture is 0.232 (*p* = 0.167), indicating a non‐significant relationship, which suggests that the relationship between pre‐lecture and post‐lecture scores is not linear or predictable based on pre‐lecture performance. This indicates that improvements in post‐lecture scores were not dependent on participants’ initial pre‐lecture scores. Furthermore, no associations between sex and education status were found with the improvement in test scores (*p* = 0.334, effect size [partial eta squared]: 0.97).

Fourteen participants believed that eating meat was unethical or immoral with no differences between the three diet groups (*p* = 0.254). Before the lecture, 18 out of 37 participants believed that a plant‐based diet could be nutritionally sufficient without supplements. This number significantly decreased to 2 participants in the follow‐up (*p* < 0.01). Regarding the influence of the presenter's credentials on participants’ trust in the information shared, 27 participants reported greater trust because the presenter was a nutrition professional. Additionally, 28 out of 37 participants stated they would consider consulting a dietitian or nutrition professional in the future. Thirty‐five out of the 37 participants stated that the lecture enabled them to make practical changes and all of them agreed that educational intervention was useful and informative because it addressed concerns specific to Adventist populations.

Five of the participants were motivated after the lecture to start using dietary supplements vitamin D, 2 started using zinc, and 3 began using curcumin for COVID‐19 prevention or treatment. Notably, the follow‐up responses revealed that 29 out of the 37 participants had experienced a shift in their perspective and views regarding the effectiveness of dietary supplements for COVID‐19 prevention and treatment after the lecture. At baseline, out of 37 participants, nearly 60% (*n* = 22) held the belief that herbal or dietary supplements were safer than vaccines and this was not dependant on gender (*p* = 0.28) or educational level (*p* = 0.41).

Lastly, the follow‐up questionnaire assessed participants’ understanding of the lecture by evaluating how well they comprehended the information presented during the session.

The response options included “all information”, “most information”, and “few information” indicating the amount of information comprehended from the lecture. All participants reported high comprehension levels as the selected responses were either the “all information” or “most information” options and none of the participants indicated that they had only grasped a small portion of the information presented.

## 4 Discussion

This study showed that an expert‐led lecture‐based educational intervention was successful in improving participants’ nutrition knowledge and resulted in belief and behavior change towards a more nutrient‐conscious approach as revealed in the follow‐up questionnaire at 1‐month post‐intervention.

The intervention was delivered by a nutrition professional, which may have contributed to its success as McGavock et al. [18] showed that expert‐led interventions targeting weight loss were associated with greater reductions in weight than interventions delivered by non‐professionals. This is confirmed by the fact, that the follow‐up questionnaire revealed that most participants trusted the presentation more due to the qualifications of the presenter.

The optimal timeframe to follow‐up with participants following educational interventions depends on the type of the intervention. This study utilized a 4‐week follow‐up, which is similar to the approach taken by Amoore et al. [19]. The relatively short follow‐up time can be justified in these instances as neither study assessed changes in disease incidence rates, biomarkers, or bodyweight—factors that typically require a longer timeline. In this regard, the 4‐week follow‐up period provided sufficient time for participants to implement some of the information presented in the lecture in regard to nutrient intake.

The information in the lecture emphasized the potential risks of nutritional deficiencies associated with plant‐based diets and the perceived benefits of supplementation, therefore, the lecture was theoretically guided by the Health Belief Model framework. A systematic review published by Jones et al. [20] showed that this health promotion model can be an effective tool to promote adherence in intervention studies and is based upon the observation that perceived barriers and benefits are the strongest predictors of behavior change. The findings of our study are in agreement with Jones et al. [20] as most participants had a change of belief concerning the adequacy of plant‐based diets and the need for supplementation.

Although the Adventists recruited for this study followed a plant‐based diet, the majority of them did not consider the consumption of meat as unethical or immoral. This differentiates plant‐based Adventists from some vegans as a literature review by Hargreaves et al. [21] suggested that most people become vegan for ethical reasons, which is most often related to animal rights concerns. Although this conflicts with the findings of Anderson and Milyavskaya [22] who showed that only 20% of the vegans participating in their study were motivated by animal welfare reasons and 42% started following the diet for health reasons. Furthermore, previous studies have shown an association between following plant‐based diets and having liberal values [23], however, Adventists are members of a conservative Protestant church [24] and hold conservative values.

Following the educational intervention, 25% of participants in this study reported starting to use dietary supplements specifically for the purpose of treating or preventing COVID‐19. These supplements included zinc, vitamin D, and curcumin, all of which have been shown to have a significant effect on immunity against COVID‐19 or aid in its treatment. The use of dietary supplements for the treatment and prevention of COVID‐19 has been extensively studied. For instance, Khasawneh et al. [25] showed in a cross‐sectional analysis that 35%, 39%, and 49% of their participants, who were living in the country of Jordan, used vitamin D, zinc, and ascorbic acid respectively for the purpose of improving their immunity against COVID‐19. Mukattash et al. [26] surveyed 2100 individuals in the Middle East and showed that 44% of participants experienced changes to their eating habits during the COVID‐19 pandemic, with 21% held the belief that dietary supplements offered protection against COVID‐19, while 45% thought they could be useful in the treatment of the infection.

These results confirm that other religious groups such as Muslims may hold similar beliefs about the usefulness and safety of natural supplements as Adventists. In addition to the data derived from observational research, interventional studies in the form of randomized controlled trials (RCTs) have been conducted to test the effect of nutritional supplements on COVID‐19‐related outcomes. Varikasuvu et al. [27] showed, based on the pooled result of RCTs that vitamin D supplementation significantly reduced COVID‐19 positivity and severity by 54%, which is thought to be attributed to its anti‐viral, immuno‐modulatory, and anti‐inflammatory effects. Similarly, evidence regarding zinc supplementation suggests that it significantly reduces COVID‐19 mortality due to its antiviral properties through the activation of immune cells such as macrophages and natural killer cells [28]. Furthermore, a meta‐analysis of clinical trials involving patients hospitalized with COVID‐19 showed that supplementation with curcumin significantly reduced the symptoms and hospital stay of patients as well as their risk of death [29]. The authors found evidence that curcumin can ameliorate the cytokine storm associated with COVID‐19 infection by significantly reducing pro‐inflammatory cytokines. On the other hand, vitamin C supplementation demonstrated no significant effect on the length of hospitalization in a meta‐analysis of 6 RCTs [30].

In addition to dietary supplements, plant‐based diets have been shown to lower the risk of COVID‐19 infection [31]. Some of the biological mechanisms involve the presence of polyphenols in plant foods, which are abundant in plant‐based diets, and have been shown to exert anti‐microbial properties. Furthermore, plant‐based diets may also contain high amounts of antioxidants such as vitamins E and C that can improve immune function and are associated with a lower risk of obesity, which is an important risk factor for COVID‐19 infection [32]. On the other hand, plant‐based diets may contain low amounts of vitamin B12, therefore, the European Food Safety Authority (EFSA) advises the use of biomarkers such as serum cobalamin or holotranscobalamin to assess nutrient status [33]. The use of vitamin B12 supplements have been shown to increase serum cobalamin levels [34], and is thus an effective way of improving vitamin B12 status. Furthermore, supplementation with vitamin D has also been shown to increase vitamin D concentrations in serum as indicated by the levels of 25‐hydroxy and 1,25‐dihydroxy vitamin D [35].

## 5 Strengths and Limitations

An important strength of this study is the 100% response rate on the post‐lecture and follow‐up questionnaires. The assessments at three different timepoints add further to the strengths of this study as it allows for the investigation of the intervention's immediate and longer‐term impacts. Furthermore, the study didn't just assess the impact of the educational intervention on participants’ knowledge and beliefs, but it also assessed behavioral change at follow‐up. The use of the Health Belief Model as a guiding framework likely facilitated a more targeted and relevant educational experience, addressing specific perceptions and behaviors related to nutritional supplements. Moreover, the questionnaire was shown to have high internal consistency during the validation study, and the sample size of the study was determined a priori, ensuring that it was adequate to detect a statistically significant change in the pre‐ and post‐lecture scores, which enhances the statistical power of the study.

The results of this study have been reported using the CHERRIES checklist, which is a tool developed to improve the quality of studies utilizing online surveys. Adherence to this guideline ensures the transparency of reporting study methods and results, thereby improving the quality of the study.

The online nature of this study may be perceived as a potential limitation; however, anonymity in online surveys has been shown to reduce response bias resulting from social desirability and may reduce anxiety experienced by the participants [36].

An important limitation of this study is the generalizability of the results to non‐Adventist plant‐based populations since Adventists are a particularly health‐conscious group, which is evident by their abstinence from smoking and alcohol as well as the availability and affordability of fortified foods and vitamin supplements can vary greatly across the globe.

Moreover, the follow‐up questionnaire may be subject to self‐reporting bias as participants could under‐ or overreport behaviors based on whether they are perceived as socially desirable or not [37]. Additionally, the reliance on self‐reported data may not only reflect potential biases in reporting but also in memory; however, the 4‐week follow‐up period can be considered a relatively short period to recall information concerning whether a person has started taking a new supplement or not. Although adequately powered based on the sample size calculation, the sample size overall could be considered small. In addition, the pilot study for the validation of the questionnaire was conducted using only 6 individuals.

A further limitation of the study is the lack of socioeconomic data collected; however, a previous study found that nutritional knowledge was not significantly associated with socioeconomic status [38], whereas educational status has been positively associated with better nutritional knowledge [39]. Data concerning educational status, however, was collected in this study, but having a higher educational background (i.e., undergraduate degree or above) showed no association with the improvement in test scores. This also shows that despite the complexity of the presented information, it was understandable by all participants and not just by the more educated ones, thereby addressing another potential limitation of the study namely that the lecture material could have been too complicated or difficult to comprehend for some participants.

This is further supported by the fact that all participants indicated reported understanding all or most of the information presented during the follow‐up survey with none indicating that they only understood some parts of the lecture.

## 6 Conclusion

This lecture‐based educational intervention conducted in the United Kingdom has proven to be highly effective in enhancing the nutritional knowledge of Seventh‐day Adventists following a vegan or vegetarian diet. The findings of this study contribute to the growing body of evidence that highlights the efficacy of educational interventions in encouraging individuals to make healthier lifestyle choices including the appropriate use of dietary supplements and fortified foods, particularly among those who consume a plant‐based diet. Notably, the educational intervention not only increased participants’ knowledge but also motivated a significant proportion of them to proactively start the use of Vitamin B12 supplements in their daily routine. These findings suggest that educational interventions can play a significant role in modifying health‐related behaviors and attitudes among Seventh‐day Adventist vegans and vegetarians; however, it is important to note that further longer‐term studies are necessary to corroborate these findings and to evaluate the long‐term impact and sustainability of such educational interventions, especially in different geographic areas where dietary supplements and fortified foods may not be readily available.

## Author Contributions

**Robert K Janko:** conceptualization, writing–original draft, methodology. **Irmgard Haussmann:** supervision, formal analysis. **Ashok Patel:** writing–review and editing, supervision, methodology. All authors read and approved the final version of the manuscript.

## Ethics Statement

This study has received ethical approval from the ethical review board of Birmingham City University.

## Consent

Participants provided informed consent.

## Conflicts of Interest

The authors declare no conflicts of interest.

## Transparency Statement

The lead author Robert K. Janko affirms that this manuscript is an honest, accurate, and transparent account of the study being reported; that no important aspects of the study have been omitted; and that any discrepancies from the study as planned (and, if relevant, registered) have been explained.

## Data Availability

The data that support the findings of this study are available on request from the corresponding author (R.K.J.). The data are not publicly available due to privacy or ethical restrictions. R.K.J. had full access to all of the data in this study and takes complete responsibility for the integrity of the data and the accuracy of the data analysis.

## References

[1] M. J. Orlich , J. Sabaté , A. Mashchak , et al., “Ultra‐Processed Food Intake and Animal‐Based Food Intake and Mortality in the Adventist Health Study‐2,” The American Journal of Clinical Nutrition 115 (2022): 1589–1601, 10.1093/ajcn/nqac043. 35199827 PMC9170476

[2] D. C. McBride , K. G. D. Bailey , P. N. Landless , A. M. Baltazar , D. J. B. Trim , and G. Stele (2021). Health beliefs, behavior, spiritual growth, and salvation in a global population of Seventh‐day Adventists. Review of Religious Research. https://openurl.ebsco.com/EPDB%3Agcd%3A13%3A14997213/detailv2?sid=ebsco%3Aplink%3Ascholar&id=ebsco%3Agcd%3A154043394&crl=f.

[3] D. L. Thorpe , W. L. Beeson , R. Knutsen , G. E. Fraser , and S. F. Knutsen , “Dietary Patterns and Hip Fracture in the Adventist Health Study 2: Combined Vitamin D and Calcium Supplementation Mitigate Increased Hip Fracture Risk Among Vegans,” The American Journal of Clinical Nutrition 114, no. 2 (2021): 488–495, 10.1093/ajcn/nqab095. 33964850 PMC8435998

[4] N. S. Rizzo , K. Jaceldo‐Siegl , J. Sabate , and G. E. Fraser , “Nutrient Profiles of Vegetarian and Nonvegetarian Dietary Patterns,” Journal of the Academy of Nutrition and Dietetics 113, no. 12 (2013): 1610–1619, 10.1016/j.jand.2013.06.349. 23988511 PMC4081456

[5] A. Niklewicz , A. D. Smith , A. Smith , et al., “The Importance of Vitamin B12 for Individuals Choosing Plant‐Based Diets,” European Journal of Nutrition 62, no. 3 (2022): 1551–1559, 10.1007/s00394-022-03025-4. 36469110 PMC10030528

[6] General Conference of Seventh‐day Adventists . (2016). Seventh‐day Adventists and nutrition. In Adventist Health Ministries. https://www.healthministries.com/seventh-day-adventists-and-nutrition/.

[7] M. A. Storz , “What Makes a Plant‐Based Diet? A Review of Current Concepts and Proposal for a Standardized Plant‐Based Dietary Intervention Checklist,” European Journal of Clinical Nutrition 76, [online] (2021): 789–800, 10.1038/s41430-021-01023-z. 34675405 PMC9187516

[8] L. Cusack , C. B. Del Mar , I. Chalmers , E. Gibson , and T. C. Hoffmann , “Educational Interventions to Improve People's Understanding of Key Concepts in Assessing the Effects of Health Interventions: A Systematic Review,” Systematic Reviews 7, no. 1 (2018): 68, 10.1186/s13643-018-0719-4. 29716639 PMC5930693

[9] E. Galvin , B. Gavin , K. Kilbride , S. Desselle , F. McNicholas , S. Cullinan , and J. Hayden , “The Use of Telehealth in Attention‐Deficit/Hyperactivity Disorder: A Survey of Parents and Caregivers,” European Child & Adolescent Psychiatry 33 (2024): 4247–4257, 10.1007/s00787-024-02466-y. 38753037 PMC11618160

[10] T. Chiba , E. Kobayashi , T. Okura , M. Sekimoto , H. Mizuno , M. Saito , and K. Umegaki , “An Educational Intervention Improved Knowledge of Dietary Supplements in College Students,” BMC Public Health 20 (2020): 633, 10.1186/s12889-020-08786-3. 32381078 PMC7204311

[11] L. M. Kent , D. P. Morton , E. J. Ward , et al., “The Influence of Religious Affiliation on Participant Responsiveness to the Complete Health Improvement Program (CHIP) Lifestyle Intervention,” Journal of Religion and Health 55, no. 5 (2015): 1561–1573, 10.1007/s10943-015-0141-3. PMC4956692 26472654

[12] N. K. Dhand and M. S. Khatkar (2014). *Statulator: An online statistical calculator. Sample size calculator for comparing two paired means*. http://statulator.com/SampleSize/ss2PM.html.

[13] E.‐J. Ha and N. Caine‐Bish , “Interactive Introductory Nutrition Course Focusing on Disease Prevention Increased Whole‐Grain Consumption by College Students,” Journal of Nutrition Education and Behavior 43, no. 4 (2011): 263–267, 10.1016/j.jneb.2010.02.008. 21419709

[14] R. K. Janko , I. Haussmann , and A. Patel , “A Longitudinal Investigation of the Prevalence and Incidence of Self‐Reported COVID‐19 Disease and the Pandemic's Impact Among Seventh‐day Adventist and Non‐Adventists Living in the UK,” Journal of Religion and Health (2024): 10.1007/s10943-024-02129-x. PMC11845397 39285080

[15] M. Conner and P. Norman (2017). Health Belief Model ‐ An overview. In ScienceDirect. https://www.sciencedirect.com/topics/medicine-and-dentistry/health-belief-model.

[16] M. Tannenbaum and S. Sebastian , “Levels of Evidence in Medical Research,” openmd.com (2021), https://openmd.com/guide/levels-of-evidence.

[17] C. R. Brydges , “Effect Size Guidelines, Sample Size Calculations, and Statistical Power in Gerontology,” Innovation in Aging 3, no. 4 (2019): igz036, 10.1093/geroni/igz036. 31528719 PMC6736231

[18] J. McGavock , B. F. Chauhan , R. Rabbani , et al., “Layperson‐Led vs Professional‐Led Behavioral Interventions for Weight Loss in Pediatric Obesity,” JAMA Network Open 3, no. 7 (2020): e2010364, 10.1001/jamanetworkopen.2020.10364. 32658289 PMC7358915

[19] B. Y. Amoore , P. K. Gaa , A. Amalba , and V. Mogre , “Nutrition Education Intervention Improves Medical Students’ Dietary Habits and Their Competency and Self‐Efficacy in Providing Nutrition Care: A Pre, Post and Follow‐Up Quasi‐Experimental Study,” Frontiers in Nutrition 10 (2023): 1063316, 10.3389/fnut.2023.1063316. 36937356 PMC10019772

[20] C. J. Jones , H. Smith , and C. Llewellyn , “Evaluating the Effectiveness of Health Belief Model Interventions in Improving Adherence: A Systematic Review,” Health Psychology Review 8, no. 3 (2013): 253–269, 10.1080/17437199.2013.802623. 25053213

[21] S. M. Hargreaves , A. Raposo , A. Saraiva , and R. P. Zandonadi , “Vegetarian Diet: An Overview Through the Perspective of Quality of Life Domains,” International Journal of Environmental Research and Public Health 18, no. 8 (2021): 4067, 10.3390/ijerph18084067. 33921521 PMC8069426

[22] J. Anderson and M. Milyavskaya , “Going Vegan or Vegetarian: Motivations & Influences,” Diet, (2021), https://www.wellbeingintlstudiesrepository.org/hw_diet/10/.

[23] C. J. Hopwood , W. Bleidorn , T. Schwaba , and S. Chen , “Health, Environmental, and Animal Rights Motives for Vegetarian Eating,” PLoS One 15, no. 4 (2020): e0230609, 10.1371/journal.pone.0230609. 32240198 PMC7117663

[24] R. L. Phillips , F. R. Lemon , W. L. Beeson , and J. W. Kuzma , “Coronary Heart Disease Mortality Among Seventh‐Day Adventists With Differing Dietary Habits: A Preliminary Report,” The American Journal of Clinical Nutrition 31, no. 10 (1978): S191–S198, 10.1093/ajcn/31.10.s191. 707372

[25] R. A. Khasawneh , S. F. Al‐Shatnawi , H. Alhamad , and K. A. Kheirallah , “Perceptions Toward the Use of Over‐The‐Counter Dietary Supplements During the Coronavirus Disease 2019 Pandemic: A Cross‐Sectional Study of the General Public in Jordan,” Health Science Reports 5, no. 4 (2022): e716, 10.1002/hsr2.716. 35844824 PMC9273937

[26] T. L. Mukattash , H. Alkhalidy , B. Alzu'bi , et al., “Dietary Supplements Intake During the Second Wave of COVID‐19 Pandemic: A Multinational Middle Eastern Study,” European Journal of Integrative Medicine 49 (2022): 102102, 10.1016/j.eujim.2022.102102. 35039757 PMC8754456

[27] S. R. Varikasuvu , B. Thangappazham , A. Vykunta , et al., “COVID‐19 and Vitamin D (Co‐VIVID Study): A Systematic Review and Meta‐Analysis of Randomized Controlled Trials,” Expert Review of Anti‐Infective Therapy 20, no. 6 (2022): 907–913, 10.1080/14787210.2022.2035217. 35086394 PMC8862170

[28] S.‐A. Tabatabaeizadeh , “Zinc Supplementation and COVID‐19 Mortality: A Meta‐Analysis,” European Journal of Medical Research 27, no. 1 (2022): 70, 10.1186/s40001-022-00694-z. 35599332 PMC9125011

[29] A. Vahedian‐Azimi , M. Abbasifard , F. Rahimi‐Bashar , et al., “Effectiveness of Curcumin on Outcomes of Hospitalized COVID‐19 Patients: A Systematic Review of Clinical Trials,” Nutrients 14, no. 2 (2022): 256, 10.3390/nu14020256. 35057437 PMC8779570

[30] D. Rawat , A. Roy , S. Maitra , A. Gulati , P. Khanna , and D. K. Baidya , “Vitamin C and COVID‐19 Treatment: A Systematic Review and Meta‐Analysis of Randomized Controlled Trials,” Diabetes & Metabolic Syndrome 15, no. 6 (2021): 102324, 10.1016/j.dsx.2021.102324. 34739908 PMC8552785

[31] A. Papadaki , E. M. Coy , D. A. Anastasilakis , N. Peradze , and C. S. Mantzoros , “The Role of Plant‐Based Dietary Patterns in Reducing COVID‐19 Risk And/Or Severity in Adults: A Systematic Review and Meta‐Analysis of Observational Studies,” Clinical Nutrition 43, no. 7 (2024): 1657–1666, 10.1016/j.clnu.2024.05.033. 38810425

[32] A. H. A. Morais , J. S. Aquino , J. K. Da silva‐Maia , S. H. L. Vale , B. L. L. Maciel , and T. S. Passos , “Nutritional Status, Diet and Viral Respiratory Infections: Perspectives for Severe Acute Respiratory Syndrome Coronavirus 2,” British Journal of Nutrition 125 (2020): 851–862, 10.1017/S0007114520003311. 32843118 PMC7542326

[33] S. Fernandes , L. Oliveira , A. Pereira , et al., “Exploring Vitamin B12 Supplementation in the Vegan Population: A Scoping Review of the Evidence,” Nutrients 16, no. 10 (2024): 1442, 10.3390/nu16101442. 38794680 PMC11124153

[34] A. Gallego‐Narbón , B. Zapatera , I. Álvarez , and M. P. Vaquero , “Methylmalonic Acid Levels and Their Relation With Cobalamin Supplementation in Spanish Vegetarians,” Plant Foods for Human Nutrition 73, no. 3 (2018): 166–171, 10.1007/s11130-018-0677-y. 29971679

[35] A. Khodadadiyan , M. Rahmanian , D. Shekouh , et al., “Evaluating the Effect of Vitamin D Supplementation on Serum Levels of 25‐hydroxy Vitamin D, 1,25‐dihydroxy Vitamin D, Parathyroid Hormone and Renin–Angiotensin–Aldosterone System: A Systematic Review and Meta‐Analysis of Clinical Trials,” BMC Nutrition 9, no. 1 (2023): 132, 10.1186/s40795-023-00786-x. 37968749 PMC10652523

[36] A. Joinson , “Social Desirability, Anonymity, and Internet‐Based Questionnaires,” Behavior Research Methods, Instruments, & Computers: A Journal of the Psychonomic Society, Inc 31, no. 3 (1999): 433–438, 10.3758/bf03200723. 10502866

[37] C. A. Latkin , C. Edwards , M. A. Davey‐Rothwell , and K. E. Tobin , “The Relationship Between Social Desirability Bias and Self‐Reports of Health, Substance Use, and Social Network Factors Among Urban Substance Users in Baltimore, Maryland,” Addictive Behaviors 73 (2017): 133–136, 10.1016/j.addbeh.2017.05.005. 28511097 PMC5519338

[38] J. Yu , X. Han , H. Wen , J. Ren , and L. Qi , “Better Dietary Knowledge and Socioeconomic Status (SES), Better Body Mass Index? Evidence from China—An Unconditional Quantile Regression Approach,” Nutrients 12, no. 4 (2020): 1197, 10.3390/nu12041197. 32344738 PMC7231000

[39] M. Lane , E. M. Barrett , A. O'Higgins , L. Mullaney , M. J. Turner , and D. McCartney , “The Relationship Between Socioeconomic Status and Nutritional Knowledge in Women During Pregnancy,” Proceedings of the Nutrition Society 72, no. OCE3 (2013): E162, 10.1017/s0029665113001857.

